# Anti-Growth and Anti-Metastatic Potential of Raw and Thermally Treated *Eragrostis tef* Extract in Human Cancer Cells

**DOI:** 10.3390/nu16162612

**Published:** 2024-08-08

**Authors:** Jina Seo, Hwa Jin Lee, Jihyeung Ju

**Affiliations:** 1 Department of Food and Nutrition, Chungbuk National University, 1 Chungdae-ro, Cheongju 28644, Chungbuk, Republic of Korea; jinaseo0725@naver.com; 2 School of Industrial Bio-Pharmaceutical Science, Semyung University, 65 Semyung-Ro, Jecheon 27136, Chungbuk, Republic of Korea; hwalee@semyung.ac.kr

**Keywords:** teff extract, thermal treatment, cancer, cell growth, metastasis

## Abstract

Teff (*Eragrostis tef*), a gluten-free cereal crop cultivated originally in Northeast Africa, is increasingly utilized due to its nutritional and health benefits. The aim of the present study was to investigate the effects of ethanol extract obtained from raw and thermally treated teff, referred to as RTE and TTE, respectively, on uncontrolled growth and activated metastasis using human cancer cell lines. Both RTE and TTE contained flavones, such as orientin (luteolin 8-C-glucoside) and vitexin (apigenin 8-C-glucoside), and phenolic acids, such as protocatechuic acid and *p*-coumaric acid. TTE showed higher total phenol, protocatechuic acid, and *p*-coumaric acid contents, but lower orientin content compared to RTE. RTE and TTE significantly suppressed cell growth of H1299 human lung cancer cells, with TTE exhibiting more pronounced effects than RTE, while both extracts had only minimal effects on the growth of non-malignant human umbilical vein endothelial cells. The growth-inhibitory activities of RTE and TTE in H1299 cells were associated with apoptosis induction and cell cycle arrest at the G2/M phase. TTE produced an additional effect on inducing cell cycle arrest at the S phase in H1299 cells, potentially contributing to its stronger growth-inhibitory effects. Moreover, both RTE and TTE effectively inhibited key events in metastasis, such as invasion, migration, and adhesion, in H1299 cells under non-cytotoxic conditions, with TTE showing stronger effects. In HCT116 human colon cancer cells, a similar pattern of inhibition was demonstrated against the metastatic events, accompanied by reduced levels of matrix metalloproteinase-2 and -9. Our results indicate that teff extracts exhibit in vitro anti-growth and anti-metastatic activities, which are enhanced by thermal treatment of teff.

## 1. Introduction

Teff (*Eragrostis tef*) is a long-cultivated cereal crop native to Northeast Africa where it serves as a staple food source [1]. In comparison to wheat and corn, teff has been reported to have higher levels of essential amino acids, calcium, iron, and zinc. It is also notable for its dietary fiber content, surpassing that of sorghum and brown [1,2]. It is a small-grained grass and typically consumed in its whole grain form, preserving the vital nutritional and functional components found in the germ and bran [1], which offers a significant advantage with the increasing recognition of the health benefits associated with whole grain consumption [3]. Moreover, teff has gained attention as a desirable gluten-free alternative to grains containing gluten, making it useful for developing gluten-free food products for individuals with gluten intolerance [4]. Due to such nutritional and health benefits of teff, its cultivation and consumption have expanded globally, reaching regions such as the United States, India, Australia, and Southwest Europe [1,5].

Cancer is a major global health concern, with lung and colorectal cancers leading to significant morbidity and mortality. Advancements in the early detection and treatment of these cancers have contributed to a decline in mortality rates [6]. However, there remains a critical need for effective anti-cancer agents with low toxicity due to the persistent challenges arising from the side effects associated with cancer treatment [7]. Considerable attention has been focused on exploring natural and dietary plant sources as potential cancer preventive and therapeutic agents that can effectively target distinct properties of cancer at different stages while exhibiting fewer side effects compared to synthetic anticancer drugs [8].

Previous phytochemical studies have reported teff grains as rich sources of phenolic compounds, including gallic acid, ferulic acid, catechin, and rutin [1,5,9,10,11,12]. Research on the biological activities of teff have primarily focused on its antioxidant activities [9,10,11,12]. Only a limited number of studies have reported its beneficial effects on hyperlipidemia, hyperglycemia, and glucose metabolism [13,14]. It is noteworthy that teff grains, like other types of grains, are commonly subjected to thermal treatment before consumption, which underscores the importance of investigating the changes in their biological activities resulting from thermal treatment. Several studies have examined the impact of different types of thermal treatment on teff, with particular focus on alterations in the antioxidant content and activities [5,15,16,17]. In a recent study, we reported the inhibitory effect of both raw and thermally treated teff on the growth of colon cancer cells [17]. The objective of the current study was to further explore potential inhibitory activities of teff against key characteristic properties of cancer cells, including uncontrolled growth and increased metastasis, and to compare the effects of raw and thermally treated teff in two different human cancer cell lines. This study was expected to expand our understanding of in vitro anti-cancer activities of teff and the influence of thermal treatment of teff on these activities.

## 2. Materials and Methods

### 2.1. Materials

Orientin and vitexin were supplied from Chemfaces (Wuhan, China). RPMI and Macoy’s 5A cell culture media were sourced from Gibco (Rockville, MD, USA). Fetal bovine serum (FBS) and streptomycin/penicillin antibiotics were obtained from Thermo Scientific (Logan, UT, USA) and Welgene Inc. (Daegu, Republic of Korea), respectively. Endothelial basal medium was purchased from Lonza (Walkersville, MD, USA). Cell culture plates and transwell chambers were purchased from Corning Inc. (New York, NY, USA). Enzyme-linked immunosorbent assay kits for matrix metalloproteinases-2 and -9 (MMP-2 and -9) were sourced from Koma Biotech Inc (Seoul, Republic of Korea). LC grade solvents were purchased from Fisher Scientific (Pittsburg, PA, USA). All other materials, unless mentioned otherwise, were sourced from Sigma-Aldrich (St. Louis, MO, USA).

### 2.2. Preparation of Raw and Thermally Treated Teff Extract

Teff (*Eragrostis tef*, originated from Djibouti) grain was purchased online as a commercial product. It was rinsed with distilled water and dried using autoclaved tissue paper to remove any excess moisture. Thermally treated teff was prepared by dry roasting at 175 °C for 10 min in a household pan without adding oil, as described previously [17]. The raw teff, without subjecting any thermal treatment, and the thermally treated teff were subsequently milled and lyophilized (PH1316, IshinBioBase, Yangju, Republic of Korea). The resulting dried samples were reconstituted with 80% ethanol in a 50-fold volume and vigorously vortexed for 24 h. After centrifugation at 2500× *g* for 5 min (A320101, Gyrozen, Daejeon, Republic of Korea), the supernatant was taken, followed by evaporation of the solvent using a centrifugal vacuum concentrator (NB-503CIR, N-bioteck, Bucheon, Republic of Korea). The resulting solid residue, denoted as raw teff extract (RTE) and thermally treated teff extract (TTE), was weighed to calculate the extraction yield (3.8% and 2.9%, respectively) and then stored at −70 °C until needed for further use.

### 2.3. Determination of Total Phenolic Contents

Total phenolic contents (TPC) were assessed spectrophotometrically, following a protocol with slight modifications from previous studies [17]. Briefly, the mixture containing 60 μL of RTE and TTE (reconstituted with ethanol and then further diluted to a concentration of 25 mg/mL), along with 30 μL of Folin Ciocalteu’s phenol reagent, and 200 μL of 7.5% sodium carbonate, underwent a reaction for 30 min at room temperature. The absorbance was measured at 650 nm using a plate reader (Bio-Rad Laboratories, Hercules, CA, USA). TPC was determined as mg gallic acid equivalent (GAE) per gram of dried extract (mg GAE/g dried extract).

### 2.4. High-Performance Liquid Chromatography Analysis

Individual phenolic compounds in RTE and TTE were analyzed using an Agilent 1260 Infinity high-performance liquid chromatography (HPLC) system with a diode array detector (DAD) (Agilent Technologies, Santa Clara, CA, USA). Compounds were separated on an Eclipse plus C18 column (250 mm × 4.6 mm, 5 μm, Agilent) at 30 °C with an injection volume of 10 μL. The mobile phases consisted of 0.01% trifluoroacetic acid in water (solvent A) and 0.01% trifluoroacetic acid in acetonitrile (solvent B) with gradient system: 5% solvent B at 0–5 min, 8.5% at 5–12 min, 21% at 12–35 min, 50% at 35–40 min, and 90% at 40–45 min. The mobile phases were flowed at a rate of 0.8 mL/min, and the detection was performed at 275 nm. Individual peaks were identified by comparing retention times and using spiking methods with authentic standards. Identified compounds were quantified by integrating the peak area and using the calibration curve for each standard.

### 2.5. Cell Culture and Common Treatment Condition

H1299 human lung cancer cells and HCT116 human colon cancer cells obtained from Korean Cell Line Bank (Seoul, Republic of Korea) were cultured in RPMI and Macoy’s 5A media, respectively. Both media were enriched with 10% FBS, 0.1 mg/mL streptomycin, and 100 units/mL penicillin. Human umbilical vein endothelial cells (HUVEC) purchased from Lonza were maintained on endothelial basal medium. All of these cells were maintained at 37 °C under the condition of 95% humidity and 5% CO_2_. The stock solutions of RTE and TTE, initially prepared using dimethyl sulfoxide (DMSO) as the solvent, were diluted with respective cell culture media without FBS shortly before each treatment. The concentration of DMSO exposed to cells was kept below 0.4% (*v*/*v*).

### 2.6. Cell Viability Assay

To assess cell growth, an assay using 3-(4,5-dimethylthiazol-2-yl)-2,5-diphenyltetrazolium bromide (MTT) was employed. H1299 cells were seeded in 96-well plates at a density of 1 × 10^4^ cells per well and allowed to adhere overnight. The cells were then incubated with varying concentrations of RTE and TTE (0, 100, 250, 500, and 1000 μg/mL) in media devoid of FBS for 24 h, 48 h, and 72 h. After the medium was aspirated, the cells were exposed with MTT at a concentration of 0.5 μg/mL for 2 h. The formazan crystals, generated by viable cells through reduction in MTT, were dissolved using DMSO. The absorbance of the resulting-colored solution was determined at 540 nm using a plate reader (Bio-Rad Laboratories).

### 2.7. Flow Cytometry

To determine the distribution of the cell cycle, flow cytometry analysis was employed [18]. H1299 cells were seeded in 6-well plates at a density of 1 × 10^5^ cells per well and cultured in FBS complete media for 24 h. The cells were then deprived of FBS for another 24 h to synchronize the cell cycle. Subsequently, the cells were exposed with RTE and TTE at the concentration of 500 μg/mL in FBS complete media for 48 h. Both adherent and floating cells were combined, rinsed with PBS, and fixed using cold 70% methanol. Subsequently, the cells exposed to propidium iodide (50 μg/mL) and ribonuclease (1 μg/mL) for 30 min at 37 °C were assessed for their distribution across cell cycle phases, including sub-G1, G1/G0, S, and G2/M, using a flow cytometer (BD Biosciences, Heidelberg, Germany). Each determination involved counting a total of 10,000 cells.

### 2.8. Transwell Assay

To assess cell invasion and migration, a transwell assay was employed using transwell chambers with 8 µm pore size. For invasion assay, the chambers were coated with matrigel (1 mg/mL) for 2 h at 37 °C, while for migration assays, the intact chambers were used. H1299 and HCT116 cells were mixed in FBS-free media containing different concentrations of RTE and TTE (0, 50, and 100 µg/mL) and loaded into the chambers at a density of 1 × 10^6^ cells per well. The cells were allowed to penetrate to the chamber toward the outer well containing complete media with FBS. After 16 h (H1299) or 24 h (HCT116) of treatment, any remaining cells on the upper surface of the chamber were gently removed using cotton swabs. The cells present on the lower surface of the chamber were stained with 0.1% crystal violet (dissolved in 20% methanol) for 30 min. After washing the stained cells with PBS, the dye was released from the cells by adding 0.5% sodium dodecyl sulfate. The invasive and migratory cells were quantified spectrophotometrically using a plate reader (Bio-Rad Laboratories) at the wavelength of 540 nm.

### 2.9. Wound-Healing Assay

A scratch wound-healing assay was performed as another assay to assess cell migration, following a previously described method [18]. H1229 cells were plated in 6-well plates at a density of 5 × 10^3^ cells per well and allowed to grow until they formed a monolayer reaching approximately 70% confluence. A wound was induced by gently scraping the cell monolayer using a 2 mm wide tip. The medium was then discarded to remove detached cells. The remaining attached cells were incubated with RTE and TTE (100 µg/mL) in FBS-free media and permitted to undergo migration for 8 h. The progression of wound closure was monitored in a minimum of five different fields using i-Solution Lite software (IMT i-Solution, Burnarby, BC, Canada). The ultimate width of wound was assessed using Image-J software version 1.52 (NIH).

### 2.10. Adhesion Assay

To assess cell adhesion, an assay using gelatin- or fibronectin-coated 96-well plates was employed, following a previously described method [18]. Gelatin (0.5%) or fibronectin (1 µg/mL) was applied onto 96-well plates by incubating for 1 h at 37 °C in Hank’s buffer. The wells then underwent blocking with 0.1% bovine serum albumin for 1 h at 37 °C. H1299 and HCT116 cells were mixed in FBS complete media containing RTE and TTE at a concentration of 1000 µg/mL and plated onto 96-well plates at a density of 2 × 10^4^ cells per well. After 1 h, the media was aspirated to eliminate the cells that had not adhered. The remaining adherent cells were stained with 0.5% MTT for 2 h at 37 °C. The resulting formazan crystals were solubilized using DMSO, and the absorbance was measured at 540 nm using a plate reader (Bio-Rad Laboratories).

### 2.11. Enzyme-Linked Immunosorbent Assay

HCT116 cells were plated in 24-well plates at a density of 1 × 10^6^ cells per well and allowed to adhere overnight. Subsequently, the cells were treated with RTE and TTE at a concentration of 100 μg/mL for 24 h. Following the treatment, the cell culture media were collected and analyzed for MMP-2 and MMP-9 levels using an enzyme-linked immunosorbent assay (ELISA) kit according to the manufacturer’s protocol.

### 2.12. Statistical Analyses

The results were expressed as mean ± SEM of at least triplicate measurements. To assess differences between the two groups, a two-tailed Student *t*-test was conducted. To determine differences among multiple groups, one-way ANOVA followed by Duncan’s multiple range test as a post-hoc analysis was performed. To examine the time- and dose-dependence of the observed effects, regression analysis was employed. Statistical significance was indicated by a *p*-value less than 0.05.

## 3. Results

### 3.1. Phenolic Contents of RTE and TTE

**Figure 1:**
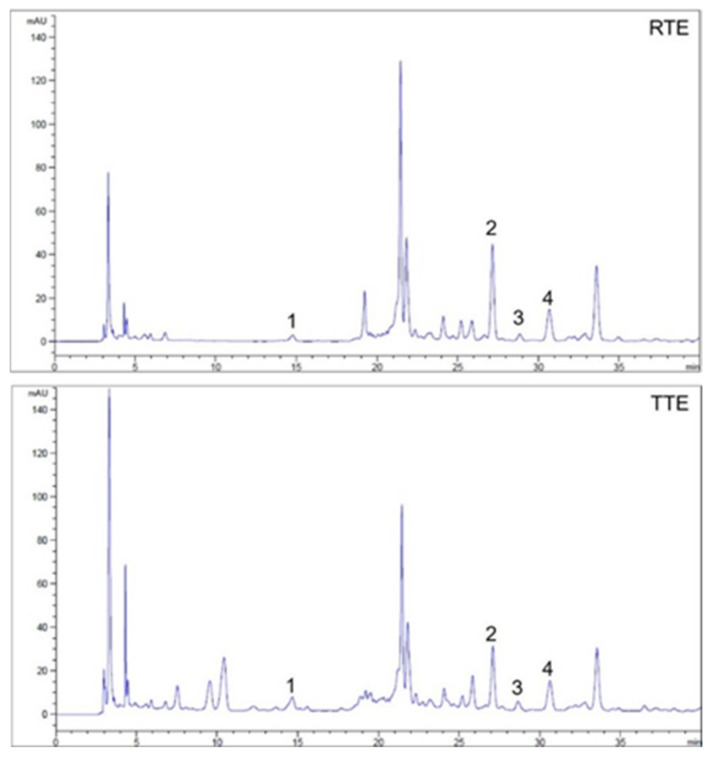
The HPLC chromatograms of RTE and TTE. 1: protocatechuic acid, 2: orientin, 3: *p*-coumaric acid, 4: vitexin.

Phenolic compounds, ubiquitously present in foods of plant origin, are recognized for their beneficial effects against a range of chronic diseases, including cancers [19]. To confirm the richness of phenolic compounds in the RTE and TTE prepared in our study, TPC in RTE and TTE were determined spectrophotometrically as an initial step. The TPC of RTE and TTE were 22.9 ± 0.5 mg GAE/g dried extract and 47.2 ± 1.7 mg GAE/g dried extract, respectively, with TTE showing a two-fold increase compared to RTE (*p* < 0.001). Subsequently, HPLC analysis was performed to determine the compositions and quantities of individual phenolic compounds present in RTE and TTE. Representative chromatograms are shown in Figure 1. Flavones such as orientin (luteolin 8-C-glucoside) and vitexin (apigenin 8-C-glucoside), as well as phenolic acids such as protocatechuic acid and *p*-coumaric acid, were detected in both RTE and TTE. To further quantify these individual phenolic compounds, both linearity and sensitivity were validated. As shown in Table 1, the linear ranges for the flavones and phenolic acids were 1–500 μg/mL and 0.1–100 μg/mL, respectively, with regression coefficients of 0.9990–1.0000. The limits of detection (LOD) and quantification (LOQ) were determined to be 0.048 to 0.134 μg/mL and 0.159 to 0.448 μg/mL, respectively. As shown in Table 2, the levels of orientin (8.160–10.719 mg/g dried extract) and vitexin (5.778–5.831 mg/g dried extract) were much higher than those of protocatechuic acid (0.291–0.866 mg/g dried extract) and *p*-coumaric acid (0.101–0.158 mg/g dried extract) in both RTE and TTE. In comparison, between RTE and TTE, TTE exhibited lower orientin content (8.160 mg/g dried extract) but higher protocatechuic acid (0.866 mg/g dried extract) and *p*-coumaric acid content (0.158 mg/g dried extract) compared to RTE (10.719 mg/g dried extract for orientin, 0.291 mg/g dried extract for protocatechuic acid, and 0.101 mg/g dried extract for *p*-coumaric acid, *p* < 0.001).

### 3.2. Effect of RTE and TTE on the Growth in H1299 Human Lung Cancer Cells

**Figure 2:**
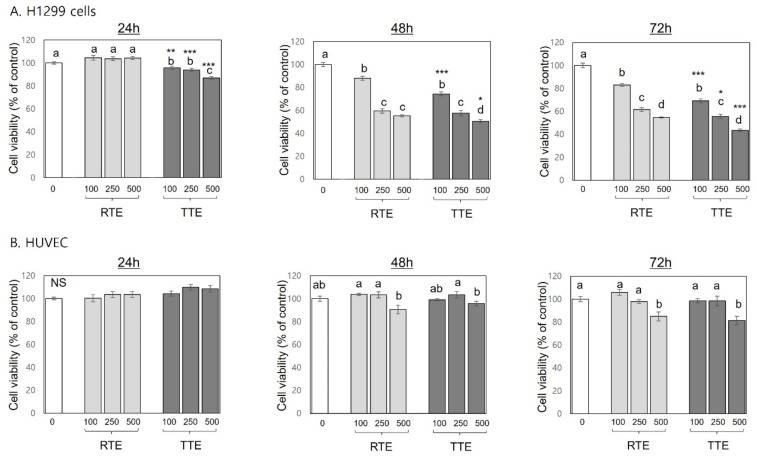
Effects of RTE and TTE on cell growth in H1299 human lung cancer cells and human umbilical vein endothelial cells. H1299 cells (**A**) and human umbilical vein endothelial cells (HUVEC) (**B**) were exposed to RTE and TTE at 0 (control, CTRL), 100, 250, and 500 μg/mL for 24 h, 48 h, and 72 h. The viability of cells was determined relative to the control. Within each treatment group (RTE and TTE), distinct letters (a–d) indicate significant differences among concentrations (*p* < 0.05). Asterisks indicate significant differences between RTE and TTE treatment at a specific concentration (* *p* < 0.05, ** *p* < 0.01, *** *p* < 0.001). NS: not significant.

Sustaining uncontrolled growth is the most fundamental hallmark exhibited by cancer cells [20]. To evaluate the inhibitory effects of RTE and TTE against cell growth on human cancer cells, cell viability assays using MTT were conducted with H1299 human lung cancer cell lines. The results were shown in Figure 2. After 24 h of treatment, RTE did not significantly decrease cell viability at the tested concentrations (100–500 µg/mL). However, prolonged treatment with RTE resulted in a more pronounced decrease in cell viability across all the concentrations, reaching 55.2–88.0% of the control at 48 h and 54.7–83.0% of the control at 72 h. Regression analysis revealed strong negative correlations between cell viabilities and treatment time or concentration (R^2^ > 0.9, *p* < 0.001), indicating a time- and dose-dependent inhibitory effect of RTE against H1299 cell growth. Treatment with TTE at all tested concentrations (100–500 µg/mL) significantly decreased the viability of H1299 cells starting at 24 h (86.9–95.7.0% of the control), and further decreases were found at 48 h (to 50.6–74.4% of the control) and 72 h (to 43.4–69.2% of the control). An inverse relationship was found between cell viability and treatment time or concentration (R^2^ > 0.9, *p* < 0.001), indicating a time- and dose-dependent growth-inhibitory effect of TTE. In the comparison between RTE and TTE, the results showed stronger growth-inhibitory effects of TTE compared to those of RTE at most concentrations and time-points (*p* < 0.05), with the exception of the 250 µg/mL at 48 h. The concentration required for half-maximal inhibition (IC_50_) for TTE was estimated to be 363.8 µg/mL at the 72 h time-point, while that for RTE was above 500 µg/mL. In non-malignant HUVEC, treatment with RTE and TTE at the concentration of 100 and 250 µg/mL showed no effects at any tested time-points (24–72 h). The highest concentration (500 µg/mL) resulted in no significant decreases in cell viability until 48 h and only a small decrease at 72 h (to 81.3–84.8% of the control).

### 3.3. Effect of RTE and TTE on Apoptosis and Cell Cycle Distribution in H1299 Cells

**Figure 3:**
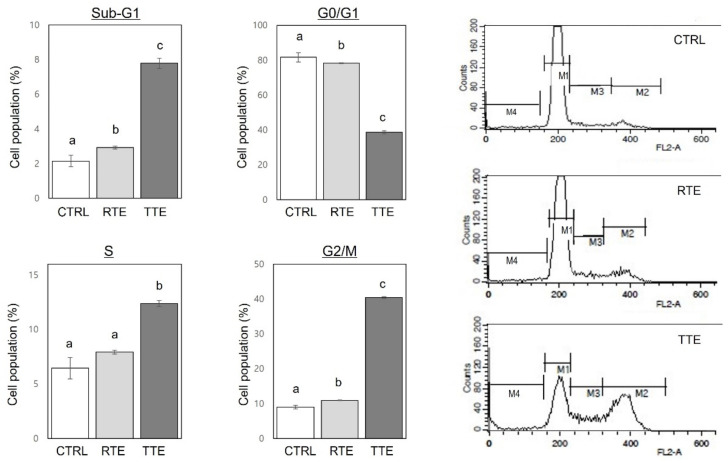
Effects of RTE and TTE on cell cycle distribution in H1299 human lung cancer cells. H1229 cells were exposed to 0 (control, CTRL) or 500 μg/mL of RTE and TTE for 48 h. The distribution of cells across sub-G1 (denoted as M4, apoptotic), G0/G1 (denoted as M1), S (denoted as M3), and G2/M (denoted as M2) phases was assessed using flow cytometry. Representative cell cycle distributions are shown in the right panel. Within each treatment group (RTE and TTE), distinct letters (a–c) indicate significant differences among concentrations (*p* < 0.05).

Impaired apoptosis and disrupted cell cycle progression are critical factors contributing to the aberrant growth of cancer cells [20]. To further investigate the characteristics of the growth-inhibitory effects of RTE and TTE (Figure 2), flow cytometry analysis was performed on H1299 cells treated with 500 µg/mL of RTE and TTE for 48 h. The analysis allowed us to detect apoptotic cells by identifying cells in the sub-G1 phase, an indicative of early apoptosis [21], and simultaneously to evaluate the distribution of cells in different phases of the cell cycle in a single analysis. As shown in Figure 3, both RTE and TTE caused significant increases in the population of cells at the sub-G1 phase, with TTE exhibiting more pronounced increases (3.6-fold of the control) compared to RTE (1.4-fold of the control). These increases in the sub-G1 phases coincided with a reduction in the population of cells at the G0/G1 phase (to 47.4–96% of the control), indicating that both RTE and TTE induced the accumulation of cell population in the sub-G1 phase. Moreover, both RTE and TTE led to an elevated percentage of cells in the G2/M phase, with TTE showing a much more prominent effect (4.5-fold of the control) compared to RTE (1.2-fold of the control). Additionally, TTE caused a significant increase in the population of cells at the S phase (1.9-fold of the control), which was not observed for RTE, indicating that TTE induced S phase arrest in addition to the apoptosis induction and cell cycle arrest at the G2/M phase.

### 3.4. Effects of RTE and TTE on Invasion in H1299 Cells

**Figure 4:**
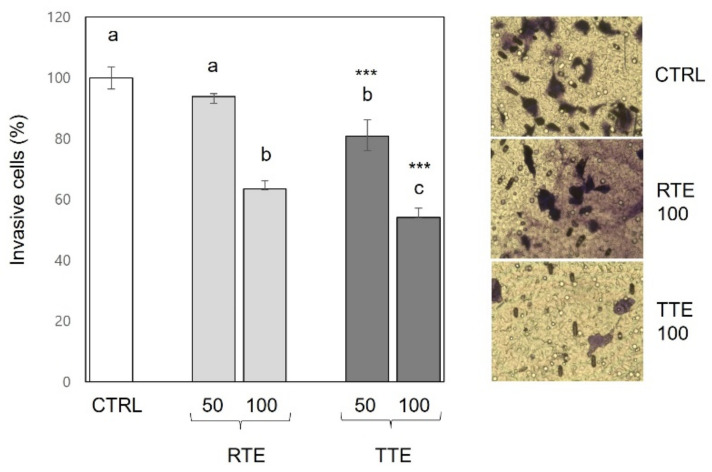
Effects of RTE and TTE on invasion in H1299 human lung cancer cells. H1299 were exposed to 0 (control, CTRL), 50, and 100 μg/mL of RTE and TTE for 16 h. The percentage of invasive cells was assessed using a transwell invasion assay, and representative images of stained invasive cells are shown in the right panel. Within each treatment group (RTE and TTE), distinct letters (a–c) indicate significant differences among concentrations (*p* < 0.05). Asterisks indicate significant differences between RTE and TTE treatment at a specific concentration (*** *p* < 0.001).

The initial step of cancer metastasis involves the invasion of cells from the primary tumor into the surrounding stroma [20]. To investigate the effects of RTE and TTE on cell invasion, a transwell assay using matrigel-coated inserts was performed. To ensure that the effects of RTE and TTE on invasion were not influenced by cytotoxicity, the assays were conducted under non-cytotoxic treatment conditions (50 and 100 μg/mL at 16 h time-point) where the cell viabilities in the treated cells were maintained at comparable levels to the control (96.8–102.6% of control). As shown in Figure 4, exposure with RTE at the 100 μg/mL concentration resulted in a significant reduction in cell invasion, lowering it to 63.4% of the control. Treatment with TTE at both 50 and 100 μg/mL concentrations led to a reduction in cell invasion to 54.1–80.9% of the control, which surpassed the inhibitory effects observed with RTE at each concentration (*p* < 0.001).

### 3.5. Effects of RTE and TTE on Migration in H1299 Cells

**Figure 5:**
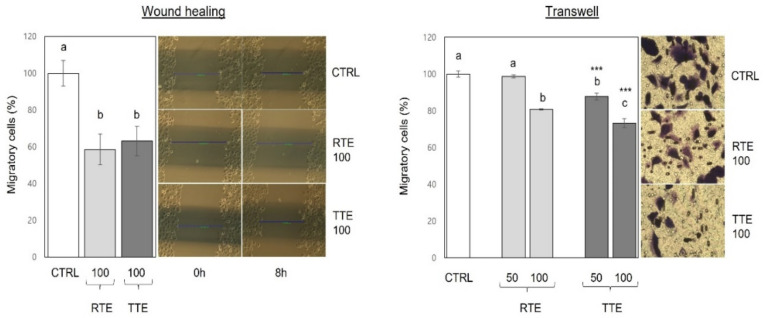
Effects of RTE and TTE on migration in H1299 human lung cancer cells. H1229 cells were exposed to 0 (control, CTRL) or 100 μg/mL of RTE and TTE in scratch wound-healing assay, and the extent of cell migration was evaluated at 8 h by measuring the closure of wounds relative to the control. H1229 were exposed to 0 (control, CTRL), 50, and 100 μg/mL of RTE and TTE for 16 h in transwell migration assay, and the extent of cell migration was determined relative to the control. Within each treatment group (RTE and TTE), distinct letters (a–c) indicate significant differences among concentrations (*p* < 0.05). Asterisks indicate significant differences between RTE and TTE treatment at a specific concentration (*** *p* < 0.001).

Cell migration is a crucial event in the metastatic cascade of cancer cells [20]. To investigate the impact of RTE and TTE on cell migration, two different assays, the wound-healing assay and transwell assay, were conducted under non-cytotoxic conditions (100 μg/mL at 8 h time-point for the wound-healing assay, 50 and 100 μg/mL at 16 h time-point for the transwell assay), and the results are shown in Figure 5. In the wound-healing assay, both RTE and TTE comparably impeded wound closure, resulting in a reduction to 58.5–63.1% of the control. However, in the transwell assay, both RTE and TTE led to a reduction in cell migration to 73.3–87.8% of the control, with TTE exhibiting stronger inhibitory effects than RTE at each concentration (*p* < 0.001).

### 3.6. Effects of RTE and TTE on Adhesion in H1299 Cells

**Figure 6:**
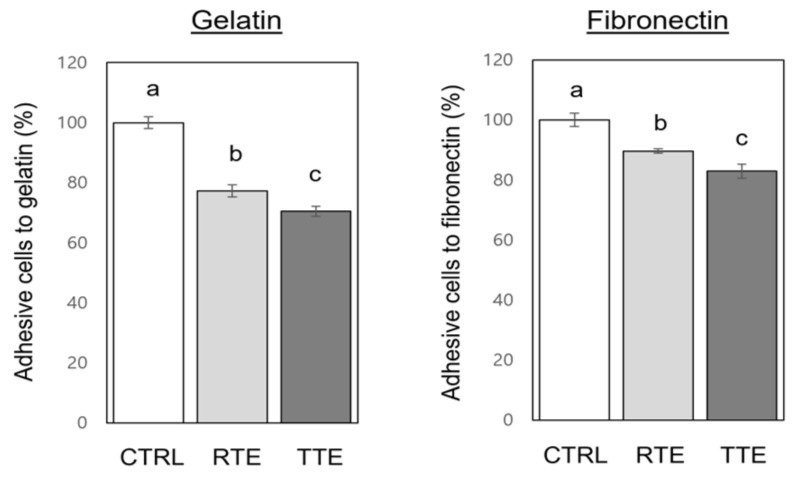
Effects of RTE and TTE on adhesion in H1299 human lung cancer cells. H1299 cells were exposed to 0 (control, CTRL) or 1000 μg/mL of RTE and TTE for 1 h, and the extent of cell adhesion to gelatin and fibronectin was determined relative to the control. Distinct letters (a–c) indicate significant differences among groups (*p* < 0.05).

Cell adhesion to extracellular matrix plays a critical role in metastasis [20]. To examine the effect of RTE and TTE on the adhesion of H1299 cells, we performed assays using plates coated with gelatin as an adhesive substrate mimicking the extracellular matrix or with fibronectin, an important glycoprotein in the extracellular matrix [22,23]. These assays were conducted under non-cytotoxic condition (1000 μg/mL at 1 h time-point). As the results show in Figure 6, treatment with RTE resulted in the inhibition of cell adhesion to both gelatin and fibronectin, reducing it to 77.3–89.6% of the control (*p* < 0.05). TTE produced a slightly stronger effect, inhibiting cell adhesion to gelatin and fibronectin to 70.5–82.9% of the control (*p* < 0.05).

### 3.7. Effects of RTE and TTE on Invasion, Migration, Adhesion, and MMP Levels in HCT116 Human Colon Cancer Cells

**Figure 7:**
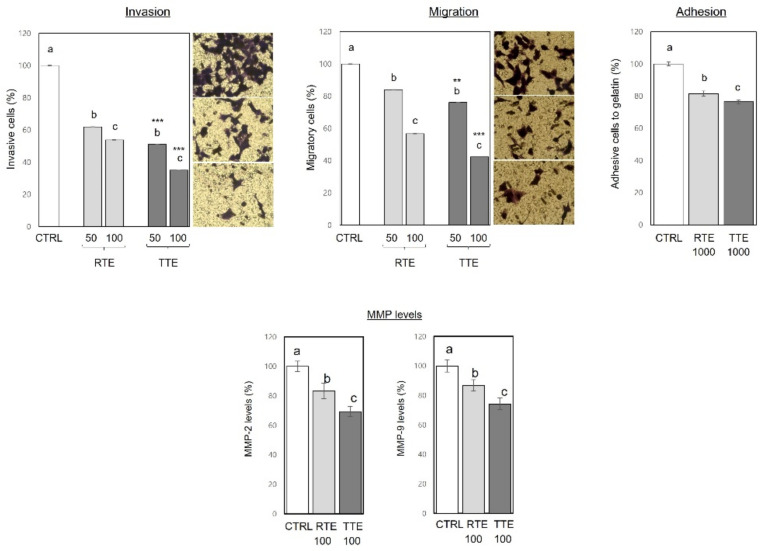
Effects of RTE and TTE on invasion, migration, adhesion, and MMP levels in HCT116 human colon cancer cells. HCT116 cells were treated with RTE and TTE under the following conditions: 0, 50, and 100 μg/mL for 24 h in transwell invasion and migration assays; 0 or 1000 μg/mL for 1 h in adhesion assay using gelatin-coated plate; and 0 or 500 μg/mL for 24 h in ELISA for MMP-2 and -9. The percentage of invasive, migratory, adhesive cells, as well as the levels of MMP-2 and -9, were determined relative to the control. Within each treatment group (RTE and TTE), distinct letters (a–c) indicate significant differences among concentrations (*p* < 0.05). Asterisks indicate significant differences between RTE and TTE treatment at a specific concentration (** *p* < 0.01, *** *p* < 0.001).

To examine if the inhibitory activities of RTE and TTE against the metastatic properties found in H1299 cells extended to other human cancer cells, HCT116 human colon cancer cell line was used, in which we previously reported growth inhibition by both RTE and TTE [17]. As shown in Figure 7, treatment with RTE and TTE resulted in inhibition of invasion (to 35.1–62.0% of the control), migration (to 42.3–84.0% of the control), and adhesion to gelatin (to 70.5–77.3% of the control) in HCT116 cells. These effects were observed under previously reported non-cytotoxic conditions (50–100 μg/mL at 24 h time-points for invasion and migration, 1000 μg/mL at 1 h time-point for adhesion) [17]. TTE exhibited slightly stronger effects compared to RTE (*p* < 0.05), with reductions to 35.1–51.0% of the control versus 53.8–62.0% of the control in invasion, 42.3–76.0% of the control versus 56.7–84.0% of the control in migration, and 76.5% of the control versus 81.6% of the control in adhesion, respectively. MMPs are a family of zinc-dependent endoproteases that degrade various proteins in the extracellular matrix. MMP-2 and MMP-9, which are gelatinases, are major contributors to invasion and further malignant progression during metastasis [24]. Both RTE and TTE at 100 μg/mL caused a significant reduction in the levels of MMP-2 to 69.3–83.2% of the control and MMP-9 to 74.2–86.9% of the control. TTE exhibited greater effects than RTE in reducing both MMP-2 and MMP-9 (*p* < 0.05).

## 4. Discussion

Cancer development is a complex and multi-step process known as carcinogenesis, involving a series of genetic and epigenetic alterations that drive the transformation of normal cells into tumorigenic and eventually malignant cells. Throughout this process, cancer cells acquire several crucial properties, including sustained growth, resisted cell death, dysregulated cell cycle, and activated metastasis [20]. Targeting these properties through dietary sources is thought to hold significant potential for developing effective and safe strategies for cancer treatment and intervention [8]. The present study aimed to investigate inhibitory activities of RTE and TTE against key properties of cancer cells using two highly proliferative and metastatic human cancer cell lines, H1299 (lung cancer cells) and HCT116 (colon cancer cells) [25,26]. The thermal treatment condition for teff employed in the present study (dry pan-roasting at 175 °C for 10 min) was selected because our previous findings demonstrated its effectiveness in enhancing antioxidant content and activities of teff extract, as well as increasing its growth-inhibitory effects in colon cancer cells, compared to other thermal treatment methods [17].

Teff grains have been documented as rich sources of phenolic compounds, with TPC ranging from 1.4 to 4.5 mg GAE/g dried grain [5,10,11,12,16]. In our study, both RTE and TTE had substantial TPC, with TTE displaying a two-fold increase over RTE (47.2 ± 1.7 mg GAE/g dried extract compared to 22.9 ± 0.3 mg GAE/g dried extract). These findings confirm the abundance of phenolic compounds in the extracts used and indicate the impact of thermal treatment on increasing TPC. Previous studies have similarly demonstrated elevated TPC in thermally treated teff [15,16,17] and millet grains [27,28,29]. Thermal application can induce transformative effects, such as denaturation of the internal grain structure, spatial expansion, and weakening of intermolecular bindings. These alterations may facilitate improved extraction of phenolic compounds, thereby collaborating for the observed elevation of TPC [30]. However, several other studies on teff grains have reported no changes in TPC upon oven roasting [16] or decreases in TPC upon oven roasting and three different hydrothermal treatments (water cooking, rice cooker, and sous-vide) [5,16], suggesting that the specific types and conditions of thermal treatment employed in each study influence the final TPC outcomes. Our results revealed that both RTE and TTE contained flavones, such as orientin (luteolin 8-C-glucoside) and vitexin (apigenin 8-C-glucoside), as well as phenolic acids, such as protocatechuic acid and *p*-coumaric acid, as individual phenolic compounds (Figure 1). These flavones and phenolic acids have been reported as major phenolic compounds in teff grain previously [1,11,31]. Orientin (8.160–10.719 mg/g dried extract) and vitexin (5.778–5.831 mg/g dried extract) were found to be more abundant than protocatechuic acid (0.291–0.866 mg/g dried extract) and *p*-coumaric acid (0.101–0.158 mg/g dried extract) in RTE and TTE (Table 2). Similarly, extractable phenolic compounds in teff grain have been reported to be almost exclusively flavones, primarily C-glycosides of luteolin and apigenin [31]. Other phenolic compounds, including gallic acid, ferulic acid, catechin, and rutin, have also been identified as major phenolics in teff grains in other previous studies [10,11]. The phenolic compositions and quantities in teff grains are likely influenced by differences in teff varieties and extraction methods used [1]. Our results showed increased levels of protocatechuic and *p*-coumaric acids but decreased levels of orientin in TTE compared to RTE (Table 2). Similarly, flavone glycosides, such as orientin, isoorientin, vitexin, and isovitexin, have been shown to decrease during thermal processing of bamboo leaves due to deglycosylation of flavonoid glycosides, yielding the corresponding aglycons [32]. The observed increases in phenolic acids in TTE might be associated with the alteration in flavone glycosides in TTE [30]. Notably, several unidentified peaks between 3 and 12 min increased, while those at 19.2 min and 21.4 min decreased in TTE (Figure 1), warranting further research on the chemical characterization of compounds resulting from thermal treatment of teff grain.

Sustaining uncontrolled growth is a fundamental characteristic of cancer cells. Apoptosis, a programmed cell death mechanism, and the cell cycle, which regulates cell duplication and division, play crucial roles in maintaining normal cell turnover. Dysregulation of apoptosis and cell cycle progression are critical factors underlying the aberrant growth of cancer cells [20,21]. Therefore, targeting the inhibition of cell growth by restoring apoptosis and the arresting of dysregulated cell cycle progression are considered effective strategies for preventing and treating cancers [21]. Our results revealed that both RTE and TTE effectively suppressed cell growth, as evidenced by decreased cell viability (Figure 2), induced apoptosis, as evidenced by increased cell population in the sub-G1 phase (Figure 3), and arrested cell cycle at the G2/M phase, as evidenced by increased cell population in the G2/M phase (Figure 3), in H1299 cells. The growth-inhibitory effects of RTE and TTE were much more pronounced in H1299 cells compared to HUVEC (Figure 2). These results indicate a greater responsiveness of H1299 cancer cells to RTE and TTE, suggesting their potential selective cytotoxicity towards malignant cancer cells over non-malignant cells. Notably, TTE exhibited more pronounced effects compared to RTE in terms of growth inhibition, apoptosis induction, and G2/M cell cycle arrest in H1299 cells. These results align with our previous study conducted in HCT116 colon cancer cells [17]. These present and previous findings collectively suggest that the growth-inhibitory effects of RTE and TTE are attributed to their activities to induce apoptosis and arrest cell cycle at the G2/M phase, and these effects are consistent across H1299 and HCT116 cells. Additionally, TTE exhibited an exclusive effect of inducing cell cycle arrest at the S phase in H1299 cells (Figure 3), which may contribute to its stronger growth-inhibitory activities than RTE (Figure 2). To the best of our knowledge, this study provides the first evidence of the effectiveness of RTE and TTE in inhibiting growth, inducing apoptosis, and arresting cell cycle in human lung cancer cells, as well as the enhanced activities of TTE. Our findings also validate that these effects are not limited to HCT116 cells, reported previously [17], but are replicated in H1299 cells.

Cancer metastasis, a formidable aspect of cancer progression and a major cause of cancer-related death, encompasses the detachment of neoplastic cells from the primary tumor and their establishment of secondary tumors in distant organs. The intricate process of metastasis necessitates cancer cells to invade neighboring tissues, migrate away from the primary tumor through the extracellular matrix, and adhere to other cells or surfaces. Therefore, cell invasion, migration, and adhesion represent pivotal events in the metastatic cascade [20]. Our results demonstrated the effective inhibition of invasion (Figure 4), migration (Figure 5), and adhesion (Figure 6) in H1299 cells by both RTE and TTE, with TTE exhibiting stronger efficacy. Furthermore, these effects were replicated in HCT116 cells (Figure 7). The only exception was observed in the wound-healing assay on H1299 cells, where RTE and TTE showed comparable inhibition (Figure 5). These results may be associated with the time point used in the assay (8 h), which might not have been sufficient to detect the difference in the effects between RTE and TTE. Alternatively, it is also possible that the wound-healing assay itself may not have been sensitive enough to accurately discern the distinction. Further research is required to provide a precise explanation for such a discrepancy. MMPs, particularly MMP-2 and MMP-9, play crucial roles in cancer metastasis by degrading the extracellular matrix components, which facilitates invasion and further progression [24]. Our results showed that both RTE and TTE reduced the levels of MMP-2 and MMP-9 in HCT116 cells, with TTE exhibiting greater effects (Figure 7). The pattern of reduction was consistent with the observed inhibition of invasion, migration, and adhesion. These findings suggest the anti-metastatic activities of RTE and TTE may be mediated through the reduction in MMP-2 and MMP-9. Further investigations of the detailed cellular and molecular mechanisms underlying their anti-metastatic effects are warranted. The inhibitory effects against invasion, migration, and adhesion (Figure 4, Figure 5, Figure 6 and Figure 7) were observed under treatment conditions that did not compromise cell viability (50–100 μg/mL at 8–16 h time-points in H1299 and at 24 h time-point in HCT116 for invasion and migration, 1000 μg/mL at 1 h time-point in both H1299 and HCT116), indicating their independence from the growth-inhibitory activities. Based on our current knowledge, this is the first study to report the anti-metastatic potential of teff grain and the enhanced activities resulting from thermal treatment of teff in human cancer cells.

Orientin, vitexin, protocatechuic acid, and *p*-coumaric acid identified in RTE and TTE have been previously demonstrated to possess a broad range of cancer-inhibitory activities [33,34,35,36]. Therefore, it is plausible to speculate that these phenolic components may contribute to the observed growth- and metastasis-inhibitory effects in this study. Orientin has previously been reported to exhibit growth-inhibitory effects in A549 cells, another type of human lung cancer cell line, with a concentration of 12.5 μM being effective [37]. This concentration is similar to the estimated concentration of orientin (approximately 12 μM) in the highest concentration of RTE used in our cell viability study (500 μg/mL, Figure 2). In addition, orientin has been reported to exhibit significant anti-migratory and anti-invasive activities, along with down-regulation of MMP-9, in MCF-7 breast cancer cells even at a low concentration of 5 μM (showing approximately 40% inhibition) [38], which is higher than but still relevant to the estimated concentration of orientin in RTE used in our invasion and migration study (2.4 μM orientin in 100 μg/mL RTE, Figure 5 and Figure 7). These findings lead us to speculate that orientin may contribute, at least partially, to the anti-growth and metastatic activities of RTE. Since the content of orientin is lower in TTE compared to RTE (Table 2), other constituents in TTE, such as protocatechuic acid, *p*-coumaric acid, and other unidentified degradation products, might interact with orientin and contribute to the observed inhibitory activities of TTE. Further detailed investigation is required to identify the specific major constituents and interactions most responsible for the observed inhibitory activities of RTE and TTE. Given that different types of thermal treatment under varying conditions have been shown to lead to different profiles and contents of polyphenols [5,15,16,17] and considering that only modest enhancement was found in the activities of TTE in the current study, it is necessary to determine the optimal thermal treatment conditions that can maximize the anti-cancer activities of teff.

## 5. Conclusions

In summary, both the RTE and TTE used in this study contained flavones, such as orientin (luteolin 8-C-glucoside) and vitexin (apigenin 8-C-glucoside), as well as phenolic acids, such as protocatechuic acid and *p*-coumaric acid, with TTE showing higher total phenolic, protocatechuic acid, and *p*-coumaric acid contents but lower orientin content compared to RTE. The inhibitory effects of both RTE and TTE on the growth of H1299 human lung cancer cells were attributed to their induction of apoptosis and cell cycle arrest at the G2/M. phase. TTE additionally induced cell cycle arrest at the S phase in H1299 cells, potentially contributing to its stronger growth-inhibitory activities compared to RTE. Moreover, both RTE and TTE suppressed key properties associated with cancer metastasis, such as invasion, migration, and adhesion, in both H1299 and HCT116 cells, with TTE exhibiting more pronounced effects. These findings demonstrate the anti-growth and anti-metastatic activities of teff extract used in our study and the enhancement of these effects following the thermal treatment of teff, suggesting their potential use in cancer prevention. However, it is imperative to acknowledge several limitations of our study. We focused on investigating the in vitro activities of teff extracts against key characteristics of cancer cells to provide initial insight, utilizing a limited number of cell lines and not including animal studies. Further research is needed to validate our findings across different types of cancer cell lines, including comparisons with normal cells, as well as in animal models and ultimately in human subjects. Our study investigated the effects of teff extracts as mixtures of various components, which may better represent the consumption of teff as a food in daily life. Nevertheless, identifying the specific major constituents and understanding the significant interactions among different constituents most responsible for the observed inhibitory activities of RTE and TTE are crucial, yet these aspects were not addressed in the current study. Additionally, further investigations are also required to elucidate the underlying cellular and molecular mechanisms of these inhibitory effects and to optimize thermal treatment protocol.

## Figures and Tables

**Table 1:** Linear regression data, limit of detection, and limit of quantification for phenolic compounds.

Phenolic Compounds		Linear Regression Data			LOD (μg/mL)	LOQ (μg/mL)
		Concentration Range Tested (μg/mL)	Calibration Curve	R^2^		
Flavones	Orientin	1.0–500	*y* = 5.8842*x* − 24.710	0.9990	0.134	0.448
	Vitexin	1.0–500	*y* = 4.8456*x* + 1.9802	1.0000	0.048	0.159
Phenolic acids	Protocatechuic acid	0.1–100	*y* = 19.487*x* − 3.5656	0.9999	0.048	0.160
	*p*-Coumaric acid	0.1–100	*y* = 46.571*x* + 6.8324	0.9998	0.051	0.169

*y* and *x* mean the peak area and concentration of the respective compound, respectively. LOD: limit of detection. LOQ: limit of quantification.

**Table 2:** Contents of phenolic compounds in RTE and TTE.

Phenolic Compounds		RTE	TTE
Flavones	Orientin	10.719 ± 0.118	8.160 ± 0.368 ***
	Vitexin	5.831 ± 0.110	5.778 ± 0.193
Phenolic acids	Protocatechuic acid	0.291 ± 0.004	0.866 ± 0.097 ***
	*p*-Coumaric acid	0.101 ± 0.003	0.158 ± 0.007 ***

mg/g dried extract. *** *p* < 0.001 via two-tailed *t*-test.

## Data Availability

All necessary data are included within this article. The data supporting the conclusions will be provided by the corresponding author on reasonable request.
